# Molecular Subtypes and Survival Patterns in Female Breast Cancer: Insights from a 12-Year Cohort

**DOI:** 10.3390/medicina61101858

**Published:** 2025-10-16

**Authors:** Ionut Marcel Cobec, Ingolf Juhasz-Böss, Peter Seropian, Sarah Huwer, Vlad Bogdan Varzaru, Andreas Rempen, Aurica Elisabeta Moatar

**Affiliations:** 1 Department of Obstetrics and Gynecology, Faculty of Medicine, Medical Center-University of Freiburg, 79106 Freiburg, Germany; 2 Clinic of Obstetrics and Gynecology, Klinikum Freudenstadt, 72250 Freudenstadt, Germany; 3 ANAPATMOL Research Center, Faculty of Medicine, “Victor Babes” University of Medicine and Pharmacy Timisoara, 300041 Timisoara, Romania; 4 Doctoral School, Faculty of Medicine, “Victor Babes” University of Medicine and Pharmacy Timisoara, 300041 Timisoara, Romania; 5 Clinic of Obstetrics and Gynecology, Diakoneo Diak Klinikum, 74523 Schwäbisch Hall, Germany; 6 Clinic of Internal Medicine-Cardiology, Klinikum Freudenstadt, 72250 Freudenstadt, Germany

**Keywords:** breast cancer, women, survival, tumor markers

## Abstract

*Background and Objectives:* Breast cancer is one of the most common cancers in women and the most common cause of cancer death. Hormone receptors, specifically the estrogen receptor (ER) and progesterone receptor (PR), as well as human epidermal growth factor receptor-2 (Her2), are tumor-specific markers used to guide breast cancer therapy. The purpose of this study is to evaluate the impact of tumor biology, including ER, PR, and Her2 expression, on survival in female breast cancer. *Materials and Methods:* This retrospective cohort study represents an analysis of 2016 female breast cancer cases using anonymized data. We reviewed cases of female breast cancer diagnosed from 1 January 2010 to 31 December 2021, in the Clinic of Obstetrics and Gynecology, Diakoneo Diak Klinikum Schwäbisch Hall, Germany. Data on clinical, pathology, immunohistochemistry, and follow-up characteristics were retrieved from the clinic’s database. To interpret the data, we used the software IBM SPSS Statistics 20, and, to account for multiple comparisons, we used a Bonferroni-adjusted significance level of 0.004. In the survival analysis, the Kaplan–Meier method and the log-rank test of equality of survival distributions were applied. *Results:* Among 2016 female breast cancer cases, 84.5% (1703/2016) were hormone receptor (HR)-positive. The 5-year overall survival was 0.873 (95% CI (0.851, 0.895); 99.6% CI (0.841, 0.905)) for HR-positive patients and 0.760 (95% CI (0.713, 0.807); 99.6% CI (0.691, 0.829)) for HR-negative patients (*p* < 0.001). Statistically significant differences were observed among HR+/HER2+, HR+/HER2−, HR−/HER2+, and triple-negative subtypes (*p* = 0.003). When comparing survival distributions based solely on HER2 expression (positive vs. negative), no statistically significant difference was observed (*p* = 0.29). *Conclusions:* Statistically significant differences in unadjusted overall survival distributions were observed among breast cancer molecular subtypes. HR-positive breast cancers demonstrated better overall survival than HR-negative cancers, while no statistically significant difference in unadjusted survival was observed between HER2-positive and HER2-negative groups.

## 1. Introduction

Breast cancer is the most frequent cancer diagnosed among women, being responsible for the highest number of cancer-related deaths among women all over the world [1,2].

According to the current medical guidelines, the standard therapy for breast cancer includes four main therapy options—surgery, radiotherapy, chemotherapy, and endocrine therapy [3]. The current standard of care for the treatment of breast cancer is neoadjuvant chemotherapy, which frequently uses targeted drugs, conservative surgery, adjuvant radiotherapy with or without adjuvant chemotherapy, and/or endocrine therapy [4].

Hormone receptors, specifically the estrogen receptor (ER) and progesterone receptor (PR), as well as human epidermal growth factor receptor-2 (Her2), are tumor-specific markers extensively used to guide breast cancer therapy.

According to the St. Gallen International Breast Cancer Conferences 2011 and 2013, it was agreed that the different breast cancer intrinsic subtypes can be defined not only by genetic array testing but also by an approximation to this classification that can be made by immunohistochemistry, and a definition of intrinsic subtypes of breast cancer was suggested: luminal A (ER+ and/or PR+, Ki67 low and Her2−), luminal B (ER+ and/or PR+, Ki67 high and/or Her2+), Her2-positive (ER−, PR− and Her2+), and triple-negative (ER−, PR−, Her2−) [5,6,7]. The hormone-receptor status and Her2 should be assessed in every diagnosed breast carcinoma in order to provide further information on mortality risk, therapy response, and decision-making [8]. Ki-67 is known as a marker of proliferative activity, and an increased index is associated with increased mitotic activity and less expression of ER and PR [9]. The immunohistochemical subtypes of breast cancer are a close reflection of the molecular breast cancer subtypes determined by microarray techniques [10].

In 3–4% of all women with breast cancer, germline mutations in *BRCA1* or *BRCA2* genes can be found, which are also involved in ovarian cancer [11,12,13,14]. Tumor immune microenvironment in ER+ breast cancer is influenced by *PIK3CA* mutation status [15]. The estrogen positivity in lobular, compared to ductal breast, carcinoma activates distinct signaling pathways including *PI3K/Akt/mTOR* signaling, in part through the actions of *WNT4* [16].

The molecular characteristics of each molecular subtype and biological features that determine each therapeutic approach and clinical outcome specific to the molecular subtype are global health concerns [17]. Breast cancer genomic profiling could contribute to better disease management, monitoring, and therapy response [17,18].

The Nottingham Prognostic Index can be applied in prognostication, using tumor size, grade, lymph node status, and ER, PR, and Her2 (with or without Ki67, a marker of proliferation) [19,20].

This observational study aimed to uncover underlying patterns between survival outcome and tumor biology—specifically ER, PR, and Her2 expression.

## 2. Materials and Methods

### 2.1. Study Design and Population

This retrospective cohort study analyzed 2016 female breast cancer cases using anonymized data collected at the Clinic of Obstetrics and Gynecology, Diakoneo Diak Klinikum Schwäbisch Hall, Germany. All consecutive cases of female breast cancer diagnosed between 1 January 2010 and 31 December 2021 that met the inclusion and exclusion criteria were reviewed.

As a retrospective full-cohort analysis, no formal sample size calculation was performed. Instead, the entire eligible population from the institutional database was included to ensure comprehensive coverage and maximize statistical power.

### 2.2. Inclusion and Exclusion Criteria

The study included only primary, non-metastatic invasive breast cancers. The following exclusion criteria were applied:

1. Male patients with breast cancer;
2. Relapses or metastases of previously diagnosed tumors (including de novo stage IV disease);
3. Breast carcinoma in situ;
4. Cases with unknown pathology results;
5. Patients with synchronous or metachronous bilateral primary breast cancers during the observed period;
6. Cases lacking follow-up data.

These criteria ensured that only patients with primary, non-metastatic invasive disease were analyzed.

### 2.3. Clinicopathologic Data

Data on clinical, pathology, immunohistochemistry, and follow-up characteristics were retrieved from the clinic’s database. The follow-up period was measured in days, from the date of diagnosis to the date of death or last contact. Patients were treated according to the German Breast Cancer Guidelines applicable during their diagnostic period, which included surgery, radiotherapy, chemotherapy, and endocrine therapy. However, due to anonymization, individual treatment regimens (chemotherapy, endocrine therapy, anti-HER2 therapy) and adherence data were unavailable for analysis.

### 2.4. Pathology and Immunohistochemistry

Estrogen receptor (ER) and progesterone receptor (PR) expression were evaluated using immunohistochemistry and classified as positive when ≥1% of tumor nuclei showed specific staining, following contemporaneous ASCO/CAP recommendations. Cases with <1% nuclear staining were considered negative. The category of ER-low (1–9%), introduced in more recent guidelines, was not separately reported during most of the study period.

HER2 expression was scored by immunohistochemistry (IHC) as 0, 1+, 2+, or 3+. Scores of 0 and 1+ were considered negative, and 3+ was considered positive. For borderline (2+) cases, fluorescence in situ hybridization (FISH) was performed when available to determine HER2 amplification status. Among 163 tumors with a 2+ IHC score, FISH results were available for 38 cases (10 positive, 28 negative). The remaining 125 HER2 2+ cases lacked FISH data due to archival limitations and were excluded from comparative analyses to avoid potential misclassification bias.

Data on Ki-67 were not included in the statistical analysis because of inconsistency in reporting practices over the 12-year period. Earlier reports described Ki-67 qualitatively, while later ones used variable numerical cut-offs; thus, standardization across all cases was not feasible.

### 2.5. Statistical Analysis

All statistical analyses were performed using IBM SPSS Statistics version 20 (IBM Corp., Armonk, NY, USA). Continuous variables were summarized as medians and interquartile ranges (IQRs), while categorical variables were expressed as frequencies and percentages.

The primary endpoint was overall survival (OS), defined as the time (in years) from diagnosis to death from any cause or last follow-up. Patients alive at last contact were treated as censored observations.

Survival functions were estimated using the Kaplan–Meier method, and differences between groups were assessed with the log-rank test for equality of survival distributions. To account for multiple comparisons, the significance threshold (α = 0.05) was adjusted using the Bonferroni correction, resulting in an effective alpha of 0.004 (rounded to three decimal places). Accordingly, both 95% confidence intervals (CIs) and Bonferroni-adjusted 99.6% CIs are reported for survival probabilities.

Because the dataset was anonymized and lacked detailed clinicopathologic and treatment variables, multivariable adjustment (e.g., Cox proportional hazards modeling) could not be performed. All results therefore represent unadjusted, exploratory comparisons of survival distributions across molecular subtypes.

Statistical significance was defined as a two-tailed *p* < 0.004 after Bonferroni adjustment.

## 3. Results

Baseline characteristics of the study cohort are summarized in Table 1. The median age at diagnosis was 63 years (interquartile range: 53–74 years). Most tumors were hormone receptor-positive (84.5%), and HER2 positivity (IHC 3+ or 2+/FISH+) was observed in approximately 16% of evaluable cases. The distribution of molecular subtypes was as follows: HR+/HER2− (69.7%), HR+/HER2+ (9.3%), HR−/HER2+ (5.8%), and triple-negative (9.1%).

Because the dataset was anonymized, detailed variables such as tumor grade, size, lymph node status, and comorbidities were unavailable for analysis.

The median age at diagnosis was 63 years (interquartile range: 53–74 years). The median follow-up time was 32.198 months, with a minimum of 1 day and a maximum of 4597 days, or 151.03 months or 12.59 years.

**Figure 1:**
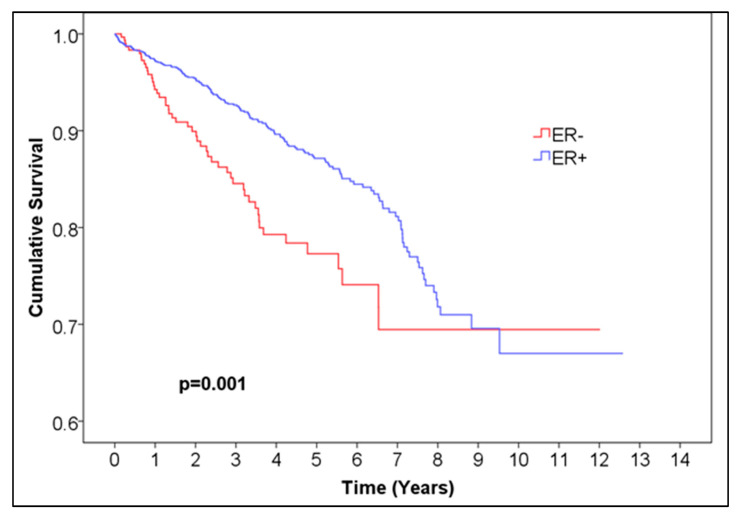
Kaplan–Meier curve of ER− vs. ER+ female breast cancer.

**Figure 2:**
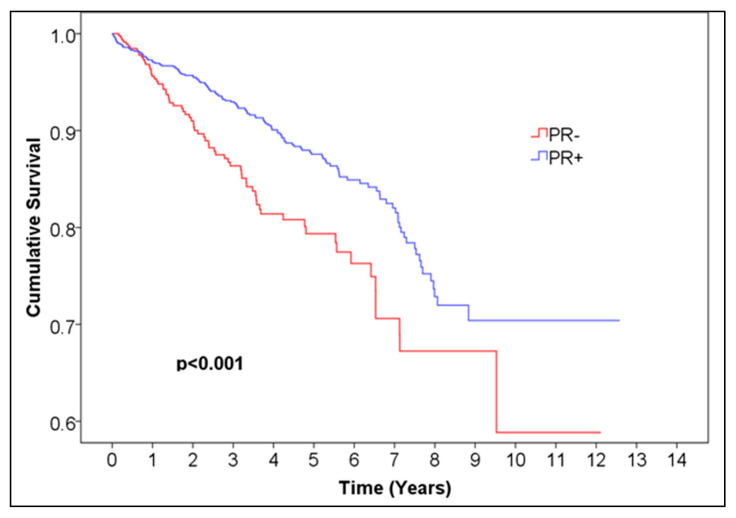
Kaplan–Meier curve of PR− vs. PR+ female breast cancer.

ER-positive tumors represented 83.5% (1683 tumors), while ER-negative tumors represented 16.5% (333 tumors). PR-positive tumors represented 73.9% (1490 tumors), while PR-negative represented 26.1% (526 tumors). Of these, 84.5% of the tumors were hormone receptor (HR)-positive. The majority of HR+ breast cancers were double positive, i.e., ER+/PR+ (1470 double positive to 233 single positive, or 83% of HR+ tumors). Her2neu immunohistochemical scores identified in our studied cohort were as follows: 0—783 cases (38.8%), 1—775 cases (38.4%), 2—163 cases (8.1%), and 3—295 cases (14.6%). FISH for Her2 amplification was performed for 38 out of 163 tumors with a Her2 score of 2: 10 were Her2-positive, and 28 were Her2 negative. Of these, 83.9% of tumors were Her2neu-negative, excluding the 125 tumors with undefined borderline Her2neu. Of the 2002 cases, 9.1% were triple-negative, while 14 cases were excluded from the analysis due to an undefined borderline Her2neu in the absence of both ER and PR (Figure 1 and Figure 2, Table 2 and Table 3).

In order to compare survival between negative and positive ER, PR, and Her2 breast cancers, we conducted a Kaplan–Meier survival analysis and the log-rank test of equality of survival distributions. The outcome variable was all-cause mortality. The time was measured in years, calculated as the number of days of follow-up divided by 365.24. Patients lost to follow-up were censored. The median survival time could not be computed, since the survival curve did not reach 0.5.

**Figure 3:**
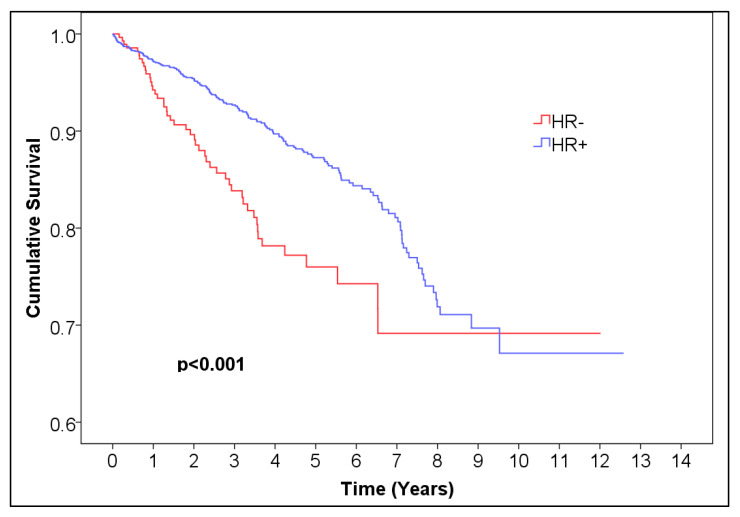
Kaplan–Meier curve of HR− vs. HR+ female breast cancer.

For ER, the 5-year overall survival was 0.872 for ER-positive patients, with 95% CI (0.85044, 0.89356) and 99.6% CI, corresponding to alpha = 0.004 (0.84034, 0.90366), and 0.773 (95% CI (0.71028, 0.83572), 99.6% CI (0.6809, 0.8651)) for ER-negative patients, *p* = 0.001 (Figure 1, Table 1). For PR, the 5-year overall survival was 0.876 (95% CI (0.85248, 0.89952), 99.6% CI (0.84146, 0.91054)) for PR-positive patients and 0.794 (95% CI (0.745, 0.843), 99.6% CI (0.72205, 0.86595)) for negative patients, *p* < 0.001 (Figure 2, Table 2). For HR, the 5-year overall survival was 0.873 (95% CI (0.85144, 0.89456), 99.6% CI (0.84134, 0.90466)) for HR-positive patients and 0.760 (95% CI (0.71296, 0.80704), 99.6% CI (0.69092, 0.82908)) for HR-negative patients, *p* < 0.001 (Figure 3, Table 4).

**Figure 4:**
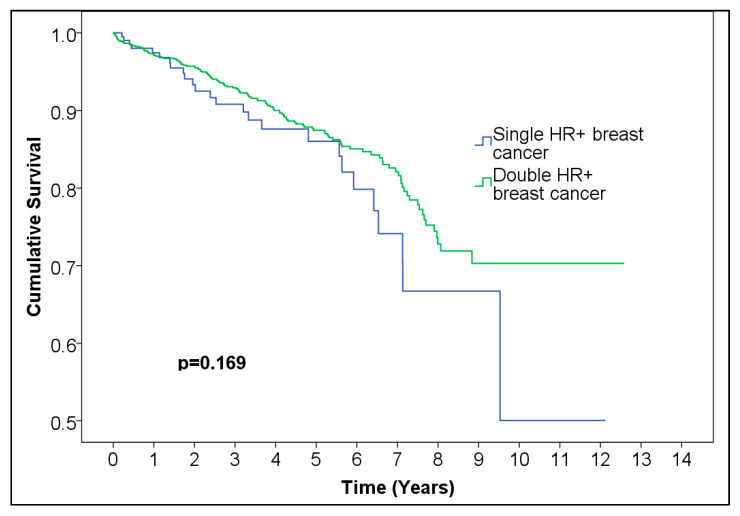
Kaplan–Meier curve of single vs. double HR+ female breast cancer.

We compared overall survival distributions of single HR-positive breast cancer (*n* = 233) and double HR-positive breast cancer (*n* = 1470) and found no statistically significant difference (*p* = 0.169) (Figure 4, Table 5).

**Figure 5:**
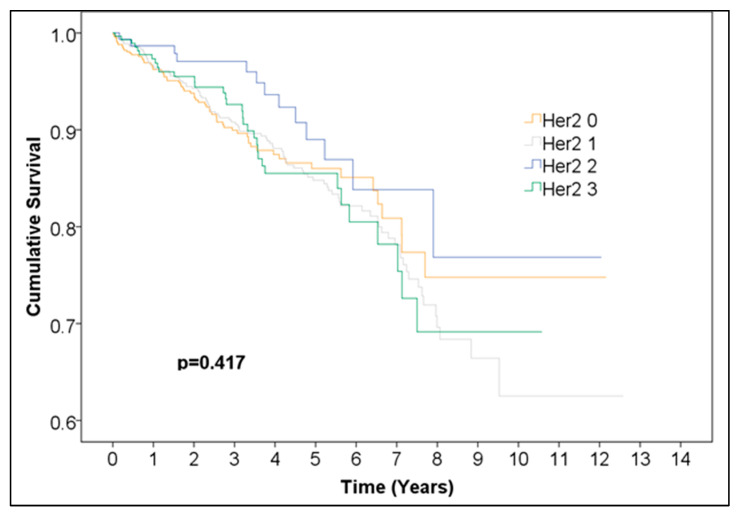
Kaplan–Meier curve of different Her2 scores in female breast cancer.

**Figure 6:**
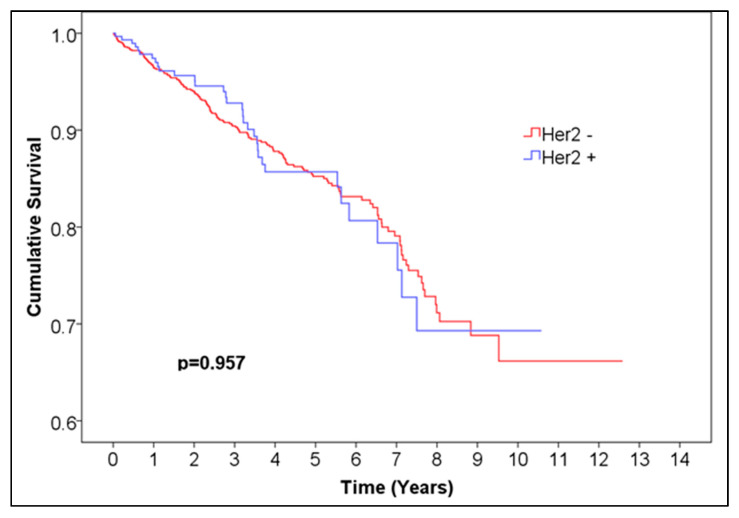
Kaplan–Meier curve of Her2− vs. Her2+ female breast cancer.

Overall survival was not significantly different between Her2 groups (0, 1, 2, 3) (Figure 5, Table 6). Additionally, we performed a Kaplan–Meier analysis for positive vs. negative Her2neu expression excluding the 125 cases with undefined borderline Her2neu scores and found no statistically significant difference between the survival curves (Figure 6, Table 7). This finding should be interpreted as indicating no statistically significant difference in observed survival distributions rather than as evidence of a lack of biological or prognostic influence.

**Figure 7:**
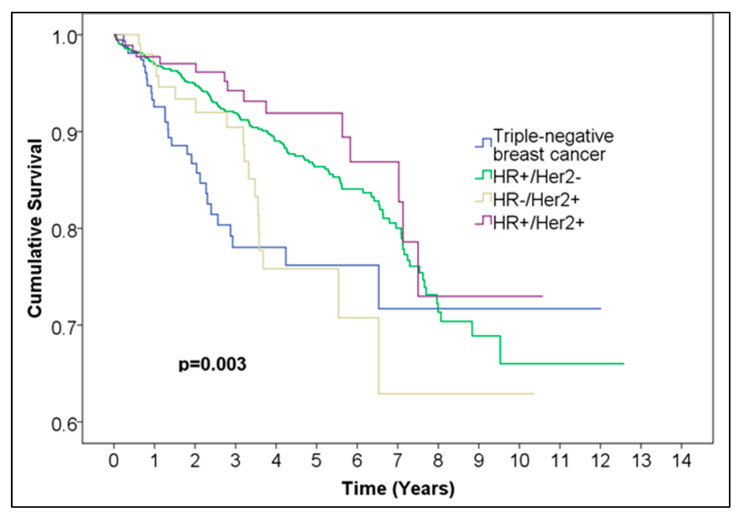
Kaplan–Meier curve of triple-negative vs. HR+/Her2− vs. HR−/Her2+ vs. HR+/Her2+ female breast cancer.

Statistically significant differences in overall survival distributions of HR+/Her2+ (188 cases), HR+/Her2− (1404 cases), HR−/Her2+ (117 cases), and triple-negative (182 cases) breast cancer were observed (*p* = 0.003) (Figure 7, Table 8). When comparing across categories, HR+/Her2+ vs. HR+/Her2− (*p* = 0.288) and HR−/Her+ vs. HR−/Her2− (triple-negative) (*p* = 0.725) indicated no statistically significant difference between the survival curves over time. Two comparisons produced *p*-values above the Bonferroni-adjusted alpha but below the uncorrected threshold of 0.05, namely HR−/Her2+ vs. HR+/Her2− (*p* = 0.036), and HR+/Her2+ vs. HR−/Her2− (triple-negative) (*p* = 0.006). The difference in overall survival between HR−/Her2− (triple-negative) and HR+/Her2− was statistically significant (*p* = 0.004).

The 5-year survival rates for each group were as follows:

- The 5-year survival HR−Her2− (triple-negative): 0.762 (95% CI (0.67772, 0.84628), 99.6% CI (0.63824, 0.88576));
- The 5-year survival HR−Her2+: 0.758 (95% CI (0.6502, 0.8658), 99.6% CI (0.5997, 0.9163));
- The 5-year survival HR+Her2−: 0.865 (95% CI (0.83952, 0.89048), 99.6% CI (0.82758, 0.90242));
- The 5-year survival HR+Her2+: 0.919 (95% CI (0.86804, 0.96996), 99.6% CI (0.84417, 0.99383)).

## 4. Discussion

The observed differences in survival between hormone receptor-positive (HR+) and hormone receptor-negative (HR−) breast cancers likely reflect both biological behavior and treatment-related effects. Because the analysis was unadjusted for tumor stage, grade, or therapy, these findings should be interpreted as descriptive associations, not independent prognostic effects.

In this retrospective, unadjusted analysis, HER2 expression did not correspond to a statistically significant difference in overall survival distributions. However, this finding should not be interpreted as implying that HER2 status has no prognostic relevance. Rather, it likely reflects the impact of effective anti-HER2 therapies and the incomplete adjudication of HER2 2+ cases. The widespread clinical adoption of targeted therapy during the study period (2010–2021) may have mitigated survival differences traditionally associated with HER2-positive disease [21].

HR+ breast cancers demonstrated better overall survival than HR− cancers, consistent with prior literature [22,23]. This difference is likely explained by endocrine sensitivity and favorable responses to hormonal therapy. We did not observe a statistically significant difference between single HR-positive and double HR-positive tumors, a finding that differs from some reports suggesting marginally better outcomes for single-positive cases [24].

When evaluating combined HR/HER2 subtypes, both HR+/HER2− and HR+/HER2+ cancers showed better survival than HR−/HER2+ and triple-negative cancers. The HR+/HER2+ subtype had the highest 5-year survival rate, which may reflect the dual benefit of hormonal and targeted biological therapies. In contrast, triple-negative and HR−/HER2+ tumors demonstrated less favorable survival, consistent with their known aggressive biology. Although these group differences reached significance at the overall level (*p* = 0.003), pairwise comparisons often fell between the nominal 0.05 threshold and the Bonferroni-adjusted α = 0.004, suggesting these results should be interpreted with caution.

Incomplete adjudication of HER2 IHC 2+ cases represents another limitation. FISH data were available for only 38 of 163 borderline cases; therefore, the remaining 125 were excluded to maintain internal validity. This may have slightly influenced the estimated HER2-positive prevalence and survival comparisons. Future analyses with complete reflex testing for all 2+ cases are warranted.

During the study period, HER2 assessment followed contemporaneous ASCO/CAP guidelines, which did not yet define the HER2-low category. Consequently, IHC 1+ and 2+/ISH– cases were not distinguished from HER2-zero tumors in a standardized manner. Given the emerging therapeutic relevance of HER2-low disease, future analyses with updated classifications are needed to better characterize this subgroup.

Although our introduction referenced St. Gallen surrogate definitions for luminal A-like and luminal B-like subtypes, consistent Ki-67 data were unavailable due to reporting variability. Earlier pathology records described Ki-67 qualitatively, while later ones used heterogeneous numerical thresholds. As a result, luminal reclassification could not be performed. Future studies with standardized Ki-67 quantification would be essential to accurately analyze luminal subtype survival.

The triple-negative breast cancer pattern was, as expected, associated with the least favorable outcomes [25]. This aligns with prior studies reporting distinct metastatic patterns by subtype: HR−/HER2+ tumors more frequently metastasize to the liver, HR+/HER2− to the lungs, and triple-negative tumors to the brain [25,26]. Bone metastases are more common in ER-positive cases, while visceral metastases are more frequent in ER-negative disease [25]. Such biological heterogeneity likely contributes to observed survival differences.

Knowledge of ER, PR, and HER2 status remains essential for optimizing systemic therapy. By assessing endocrine and HER2 sensitivity, clinicians can tailor treatment strategies and better predict therapeutic response [27]. Even tumors with <10% ER positivity may behave biologically as ER-positive and benefit from adjuvant endocrine therapy [27,28]. Adjuvant tamoxifen therapy, for example, reduces breast cancer mortality by roughly 31% at five years, with cumulative benefits extending to 15 years post-diagnosis [29]. HER2-positive and triple-negative cancers generally exhibit higher recurrence rates and lower disease-free survival, whereas HR-positive tumors have more favorable long-term outcomes [30,31,32,33,34,35].

The results of this study have direct practical relevance for clinical and pathology workflows. By confirming the significant survival advantage of hormone receptor-positive disease and the comparable survival of HER2-positive and HER2-negative subtypes under modern treatment conditions, our data underscore the ongoing importance of accurate ER, PR, and HER2 determination for therapeutic decision-making. These findings support the use of standardized immunohistochemical assessment and reporting especially in regional centers to ensure consistent identification of molecular subtypes that guide systemic therapy. Furthermore, the observed survival patterns provide a valuable reference for institutional benchmarking and the design of future multicenter outcome studies integrating treatment and molecular data.

Finally, de novo stage IV cases were excluded, thereby restricting the cohort to primary, non-metastatic invasive breast cancers. This design choice was intended to minimize survival confounding from advanced-stage disease.

### Limitations

This study has several limitations. Because some statistical tests were not fully independent, the Bonferroni correction may have been overly conservative. To address this, both the original and Bonferroni-adjusted confidence intervals were reported. The retrospective nature of the study and its reliance on an anonymized dataset mean that the analysis is exploratory rather than confirmatory.

A major limitation is the absence of clinicopathologic and treatment covariates, which precluded multivariable Cox regression analysis. Potential confounding by stage at diagnosis, tumor grade, and treatment type cannot therefore be excluded. Incomplete HER2 adjudication (particularly among IHC 2+ cases) may also have influenced classification accuracy. Because HER2-low and ER-low categories were not defined during much of the study period, these subgroups could not be analyzed separately. Inconsistent Ki-67 reporting prevented accurate luminal A/B-like classification.

Data on systemic therapy, including anti-HER2 treatment, chemotherapy, and endocrine therapy adherence, were not available. Consequently, the observed survival outcomes may reflect both biological and therapeutic factors rather than pure biomarker effects. Finally, because stage IV cases were excluded, the findings apply primarily to early and locally advanced breast cancers and do not reflect outcomes in metastatic disease.

## 5. Conclusions

Statistically significant differences were observed between the overall survival distributions of different breast cancer molecular subtypes. Hormone receptor-positive (HR+) breast cancers showed significantly better overall survival compared with hormone receptor-negative (HR−) tumors. In contrast, no statistically significant difference was found between HER2-positive and HER2-negative survival distributions in this unadjusted cohort.

These results demonstrate significant differences in unadjusted overall survival across breast cancer molecular patterns, primarily driven by hormone receptor status. However, they should be interpreted with caution, as therapy effects, treatment-era influences (including the introduction of targeted therapies for HER2-positive disease), and incomplete HER2 adjudication may have affected the observed outcomes. Future studies incorporating detailed clinicopathologic and treatment data are warranted to confirm the independent prognostic impact of these molecular markers.

## Figures and Tables

**Table 1:** Baseline characteristics of the study population (*n* = 2016). Abbreviations: HR = hormone receptor (estrogen and/or progesterone receptor); HER2 = human epidermal growth factor receptor 2; IQR = interquartile range.

Variable	Total (*n*, %)	HR+/HER2−	HR+/HER2+	HR−/HER2+	Triple-Negative (HR−/HER2−)
Number of cases	2016 (100%)	1404 (69.7%)	188 (9.3%)	117 (5.8%)	182 (9.1%)
Median age (years)	63 (IQR 53–74)	63	62	61	59

**Table 2:** Number of patients at risk of ER− vs. ER+ female breast cancer. Abbreviations: ER = estrogen receptor.

Time (Years)	0	1	2	3	4	5	6	7	8	9	10	11	12	13
Number at risk ER−	333	240	179	143	98	64	36	27	14	5	2	1	1	0
Number at risk ER+	1683	1258	999	804	612	448	280	187	95	41	18	10	7	0

**Table 3:** Number of patients at risk of PR− vs. PR+ female breast cancer. Abbreviations: PR = progesterone receptor.

Time (Years)	0	1	2	3	4	5	6	7	8	9	10	11	12	13
Number at risk PR−	526	375	272	217	147	102	63	44	20	8	5	3	2	0
Number at risk PR+	1490	1123	906	730	563	410	253	170	89	38	15	8	6	0

**Table 4:** Number of patients at risk of HR− vs. HR+ female breast cancer. Abbreviations: HR = hormone receptor.

Time (Years)	0	1	2	3	4	5	6	7	8	9	10	11	12	13
Number at risk HR−	313	225	167	132	89	57	32	25	12	4	2	1	1	0
Number at risk HR+	1703	1273	1011	815	621	455	284	189	97	42	18	10	7	0

**Table 5:** Number of patients at risk of single vs. double HR+ female breast cancer. Abbreviations: HR = hormone receptor; HR+ = estrogen and/or progesterone receptor–positive.

Time (Years)	0	1	2	3	4	5	6	7	8	9	10	11	12	13
Number at risk Single HR+ breast cancer	233	165	117	96	67	52	35	21	10	5	3	2	1	0
Number at risk Double HR+ breast cancer	1470	1108	894	719	554	403	249	168	87	37	15	8	6	0

**Table 6:** Number of patients at risk of different Her2 scores in female breast cancer. Abbreviations: HER2 = human epidermal growth factor receptor 2; IHC = immunohistochemistry.

Time (Years)	0	1	2	3	4	5	6	7	8	9	10	11	12	13
Number at risk Her2 0	783	541	402	307	204	139	74	48	24	12	5	3	1	0
Number at risk Her2 1	775	598	491	400	333	256	172	119	60	28	12	7	6	0
Number at risk Her2 2	163	135	110	98	73	48	26	18	10	1	1	1	1	0
Number at risk Her2 3	295	224	175	142	100	69	44	29	15	5	2	0	0	0

**Table 7:** Number of patients at risk of Her2− vs. Her2+ female breast cancer. Abbreviations: HER2 = human epidermal growth factor receptor 2.

Time (Years)	0	1	2	3	4	5	6	7	8	9	10	11	12	13
Number at risk Her2−	1586	1156	902	715	539	395	246	167	84	40	17	10	7	0
Number at risk Her2+	305	230	178	144	100	68	44	29	15	5	2	0	0	0

**Table 8:** Number of patients at risk of triple-negative vs. HR+/Her2− vs. HR−/Her2+ vs. HR+/Her2+ female breast cancer. Abbreviations: HR = hormone receptor (estrogen and/or progesterone receptor); HER2 = human epidermal growth factor receptor 2.

Time (Years)	0	1	2	3	4	5	6	7	8	9	10	11	12	13
Number at risk Triple-negative breast cancer	182	126	88	66	46	31	18	15	5	2	1	1	1	0
Number at risk HR+/Her2−	1404	1030	814	649	493	364	228	152	79	38	16	9	6	0
Number at risk HR−/Her2+	117	87	67	54	33	20	11	7	5	2	1	0	0	0
Number at risk HR+/Her2+	188	143	111	90	67	49	33	22	10	3	1	0	0	0

## Data Availability

Further information concerning the present study is available from the corresponding author upon reasonable request.
